# Characterizing the sectoral development of cities

**DOI:** 10.1371/journal.pone.0254601

**Published:** 2021-07-14

**Authors:** Diego Rybski, Prajal Pradhan, Shade T. Shutters, Van Butsic, Jürgen P. Kropp

**Affiliations:** 1 Potsdam Institute for Climate Impact Research—PIK, Member of Leibniz Association, Potsdam, Germany; 2 Department of Environmental Science Policy and Management, University of California Berkeley, Berkeley, CA, United States of America; 3 Complexity Science Hub Vienna, Vienna, Austria; 4 School of Complex Adaptive Systems, Arizona State University, Tempe, AZ, United States of America; 5 Global Climate Forum, Berlin, Germany; 6 Institute of Earth and Environmental Science, University of Potsdam, Potsdam, Germany; Institute for Advanced Sustainability Studies, GERMANY

## Abstract

Previous research has identified a predictive model of how a nation’s distribution of gross domestic product (GDP) among agriculture (*a*), industry (*i*), and services (*s*) changes as a country develops. Here we use this national model to analyze the composition of GDP for US Metropolitan Statistical Areas (MSA) over time. To characterize the transfer of GDP shares between the sectors in the course of economic development we explore a simple system of differential equations proposed in the country-level model. Fitting the model to more than 120 MSAs we find that according to the obtained parameters MSAs can be classified into 6 groups (consecutive, high industry, re-industrializing; each of them also with reversed development direction). The consecutive transfer (*a* → *i* → *s*) is common but does not represent all MSAs examined. At the 95% confidence level, 40% of MSAs belong to types exhibiting an increasing share of GDP from agriculture. In California, such MSAs, which we classify as part of an agriculture renaissance, are found in the Central Valley.

## Introduction

The Neolithic Revolution, the Industrial Revolution, and recent Globalization can be seen as cornerstones of human development and the evolution of cities [1]. During the Neolithic Revolution, agricultural practices were established and settlements and cities emerged. The Industrial Revolution saw the emergence of factories in cities and subsequently strong growth rates. Globalization, in turn, is characterized by pronounced services in developed countries and industrialization of transitioning and developing countries.

Thus, the historical emergence of cities can also be related to economic sectors. From a point of view of economic development one can distinguish the agricultural, industrial, and service sectors (primary, secondary, and tertiary, respectively) [2–5]. Moreover, it has been shown that the fraction of rural population statistically decreases with lower shares of GDP from agriculture [6, 7], implying that urbanization is a process that is naturally coupled with economic development.

It can be observed that at the scale of countries the three sectors replace each other following trajectories that differ considerably from country to country [7]. In early stages of development, agriculture is the most important economic activity. As economic development progresses, agriculture is replaced by a growing contribution of industry to the economy, which is then increasingly replaced by the service sector (Three-Sector Hypothesis [5, e.g.]). Some economies bypass the industrial development phase and progress directly to a service-dominated economy, such as tourism in the case of some island states. As a consequence, today’s most developed countries exhibit minor agricultural sectors [8] and service sectors of dominating economic importance. Despite a well-developed qualitative understanding of economic sectors, quantitative empirical work characterizing sectoral development is limited, especially in cities.

GDP is the most widely used measure of economic health yet it is still not well understood [9]. Despite this lack of understanding and other criticisms it is the key factor in economic policy making from local to global scales [10]. GDP is linked with nearly every United Nations Sustainable Development Goal and is the primary focus of SDG8: “Decent Work and Economic Growth” [11, 12]. Thus if GDP is to continue being used to as the primary input into economic policies it is critical to better understand its dynamics. Studies of GDP dynamics over time rarely examine the dynamics of individual components of GDP and when they do, it is almost exclusively at the national [13, e.g.] or multi-nation level, such as the European Union as a whole [14]. While studies of GDP temporal dynamics at the city level do exist, they typically focus on changes in growth rates and not on structural change [15, e.g.].

In many instances, understanding of urban dynamics has been enhanced by implementing models and methods used to understand dynamics of nations. For instance, in parallel to country-level studies, researchers have analyzed intercity trade [16], intercity relations [17], migration flows between cities [18], even migration patterns within cities [19], and food systems of cities [20]. Here, we advance this tradition by exploring the hypothesis that cities exhibit sectoral development analogous to that of countries. In the extreme idealization, villages and towns are dominated by farming of surrounding lands. In the other extreme, metropolises exhibit a much developed and diversified service economy whereas agriculture and industry are marginal. This sectoral relocation proceeds concurrently with urban growth and an increase of economic power.

We empirically study the shares of GDP in US Metropolitan Statistical Areas (MSA) and characterize their evolution during the years 2001-2019. We choose the USA because they have strong industry, large agriculture, and a mature urban system. Moreover, the MSAs represent an established definition of metropolitan areas that take economic links across administrative boundaries into account and thereby better represent functional cities than e.g. counties. We use a transition model to characterize the dynamics and by applying strict criteria on the data integrity (126 out of 384 MSAs) as well as confidence of regressions (40 out of 126 MSAs) we ensure robust results. Based on the obtained parameters we categorize the MSAs into 6 types and observe that a considerable fraction of analyzed MSAs exhibit development patterns with an increasing share of GDP from agriculture. However, we observe only 5 types among 126 MSAs and 4 types among MSAs with 95% confidence. California is an interesting case since these MSAs of the so-called agriculture renaissance are located in relatively close proximity to MSAs with concentrations of high-tech companies. We take the opportunity to address perspectives of (peri)urban agriculture.

## Dynamical Sector Transfer model

We use the *Dynamical Sector Transfer* (DST) model which was originally introduced to characterize the developmental changes in GDP composition at the country scale [7]. Here we adopt the model to the economic composition of cities. As detailed in the Materials and methods, the model describes the relocation of economic activity among the aggregated sectors *agriculture* (*a*), *industry* (*i*), and *service* (*s*), which are parameterized against the logarithm of total urban GDP (*g* = ln*G*) serving as indicator of (economic) development. The solution to the system of coupled differential equations is given by [7]

$$\begin{array}{ccc} a(g) & = & e^{-k_{1}\left(g-g_{0}\right)} \end{array}$$ (1)

$$\begin{array}{ccc} i(g) & = & \frac{\alpha k_{1}}{k_{2}-k_{1}}(e^{-k_{1}\left(g-g_{0}\right)}-e^{-k_{2}\left(g-g_{0}\right)}) \end{array}$$ (2)

$$\begin{array}{ccc} s(g) & = & 1-a(g)-i(g)\;, \end{array}$$ (3)

where *k*_1_, *k*_2_, and *α* are parameters. An additional parameter *g*_0_ emerges from integration which corresponds to the *g* at which there is solely agriculture. The transfer from agriculture is determined by *k*_1_, which for *α* = 1 goes fully to industry and for *α* = 0 goes fully to service (for 0 < *α* < 1 the transfer is split). The transfer from industry to service is determined by *k*_2_. For *k*_1_ > 0, *k*_2_ > 0, and 0 ≤ *α* ≤ 1 the transfer is directed from agriculture (via industry) to service.

## Development types and examples

**Fig 1:**
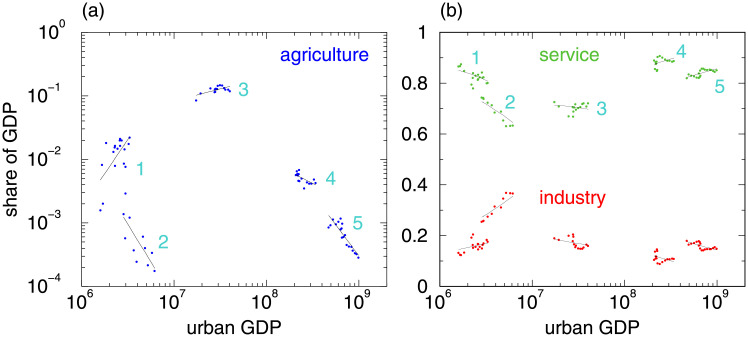
Metropolitan Statistical Area (MSA) examples of sectoral development. (a) Agriculture, *a*(*g*), as a function of the total urban GDP in log-log representation; (b) industry (bottom), *i*, and service (top), *s*, as a function of the total urban GDP in log-linear representation. The MSA are, 1: Great Falls, MT (type A^−^ [96%], GeoFIPS 24500), 2: East Stroudsburg, PA (type B [100%], GeoFIPS 20700), 3: Fresno, CA (type B^−^ [97%], GeoFIPS 23420), 4: Miami-Fort Lauderdale-Pompano Beach, FL (type C [81%], GeoFIPS 33100), and 5: Los Angeles-Long Beach-Anaheim, CA (type A [100%], GeoFIPS 31080). Percentages in square-brackets represent confidence from bootstrapping. Dots represent the data and straight lines stem from the DST model Eqs (1)–(3).

Fig 1 shows the data from example MSAs and includes curves that we obtain from non-linear curve fitting (see Materials and methods for details). Due to the small values of agricultural shares, we use double-logarithmic representation in Fig 1(a), so that Eq (1) becomes a straight line. In some MSAs the share of agricultural GDP increases. Industrial and service shares are displayed in Fig 1(b) (semi-logarithmic representation). While these examples exhibit higher shares of services, not all develop towards larger *s*. Despite some fluctuation—e.g. due to the subprime mortgage crisis 2007-2010—the regressions reasonably capture the dynamics of the urban economic systems.

Following Lutz et al. [7] we do not constrain the ranges of the 4 parameters (but find *α* > 0 in all cases). The 6 valid types of MSA economies are listed in Table 1 (see Materials and methods for details). The different types essentially stem from the parameter ranges as indicated in Table 1, e.g. when *g* − *g*_0_ < 0 is true then the negative sign in the exponentials in Eqs (1)–(3) vanishes and *a*(*g*) increases with growing *g* although *k*_1_ > 0. Type A is the consecutive, “conventional” case. With increasing *g* agriculture decreases, industry exhibits a hump, and service monotonously increases. Its counterpart type A^−^ exhibits the same shape but reversed, i.e. with increasing GDP the dynamics is towards agriculture. In type B, service and industry are swapped in the sense that service exhibits a maximum and industry keeps increasing. These MSAs can maybe understood as reindustrializing ones. Type B^−^ is the reversed counterpart. Type C is similar to type A but due to *α* > 1 for given *k*_1_ and *k*_2_ industry can reach higher shares of industry compared to type A. Past dynamics led to the high values of industry. These cases can be understood as industrial MSAs. Type C^−^ is again the corresponding counterpart.

**Table 1:** Development types of the DST model, Eqs (1)–(3).

Type	*αk*_1_ < 0	*k*_2_ < 0	(1 − *α*)*k*_1_ < 0	*g* − *g*_0_ < 0	Dynamics	valid for
A	0	0	0	0	*a* → *i* → *s*	*g*_0_ < *g*
					*a* → *s*	
B	0	1	0	0	*a* → *i* ← *s*	*g*_0_ < *g* < *g*_max_
					*a* → *s*	
C	0	0	1	0	*a* → *i* → *s*	*g*_0_ < *g*_min_ < *g*
					*a* ← *s*	
C^−^	1	1	0	1	*s* → *i* → *a*	*g* < *g*_max_ < *g*_0_
					*s* ← *a*	
B^−^	1	0	1	1	*s* ← *i* → *a*	*g*_min_ < *g* < *g*_0_
					*s* → *a*	
A^−^	1	1	1	1	*s* → *i* → *a*	*g* < *g*_0_
					*s* → *a*	

The types are given by parameter ranges. Other regions lead to impossible situations, such as *a* > 1. The types A^−^, B^−^, C^−^ are equivalent to the types A, B, C, respectively, but exhibit time reversal. ‘Dynamics’ illustrates the direction of transfer. ‘valid for’ provides the ranges of *g* for which the type is defined, whereas *g*_min_ and *g*_max_ depend on parameters.

Fig 1 shows examples of five general types of MSA sectoral development (A, B, C, B^−^, A^−^). The sixth (C^−^) does not occur. The MSAs of type A, including Los Angeles, exhibit decreasing agriculture and industry and increasing service. MSAs of type B, including East Stroudburg, PA, display decreasing agriculture and increasing industry. The Miami MSA (type C) exhibits decreasing agriculture, decreasing industry, and increasing service. Increasing agriculture can e.g. be observed for the Fresno, CA MSA (type B^−^) accompanied by decreasing industry and decreasing service. MSAs of type A^−^, like Great Falls, MT, represent the mirrored case of type A, i.e. increasing agriculture and decreasing service.

## Dominant development types

Table 2 displays the frequency of each classification type among our set of 126 MSAs. We find 59 instances of A or A^−^, 64 of B or B^−^, and 3 of C or C^−^. However, 57 out of 126, i.e. almost 50%, of the considered MSAs exhibit “time reversal” where the MSAs are experiencing an increasing agricultural sector. If we consider only those MSA where the determined type is confident at the 95% level (from bootstrapping), then 40 instances remain. More than half of them follow the consecutive development (type A) and a third belongs to type B^−^. Still 40% exhibit “time reversal” (belonging to type A^−^ or B^−^). Neither type C nor type C^−^ occur. Among the instances of type A (with 95% confidence) the parameter *α*, which splits the flows from agriculture into industry or service, roughly takes values between 0.2 and 0.5. These values closer to 0 than to 1 indicate that a considerable part of the flows goes directly to service bypassing industry.

**Table 2:** Occurrences of the development types detailed in Table 1.

Type	Count	Ave.Pop.	Count	Ave.Pop.
	all considered MSAs		95% confidence	
A	43 (34%)	772	22 (55%)	1112
B	23 (18%)	313	2 (5%)	143
C	3 (2%)	748	0 (0%)	-
C^−^	0 (0%)	-	0 (0%)	-
B^−^	41 (33%)	446	14 (35%)	436
A^−^	16 (13%)	249	2 (5%)	111

‘all considered MSAs’ refers to the entire set of 126 MSA and ‘95% confidence’ refers to only those 40 MSA where confidence from bootstrapping is at least 95%. ‘Ave.Pop.’ stands for average population of the MSA (2010) in multiple of 1,000. Almost half of the MSA are of types A^−^, B^−^, or C^−^, exhibiting increasing shares of agriculture GDP. MSAs of type A are the largest in terms of population.

Table 2 also includes the average population of the MSAs according to each type. Under the stricter 95%-confidence, the average population size of type A MSAs is more than a million, type B^−^ take the second place with less than half the size, and MSAs of types B and A^−^ are not only rare, but also small. This suggests that although other types occur, type A is to some extent a dominant one. Considering all analyzed MSAs, in terms of population C MSAs are the second largest type—although only consisting of three instances.

**Fig 2:**
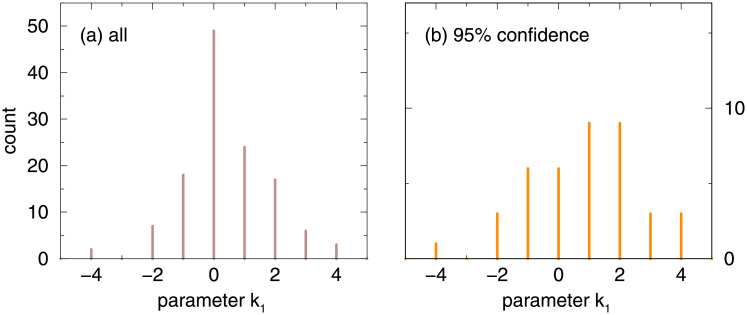
Histogram of the parameter *k*_1_ from Eq (1) for (a) all considered MSAs and (b) those MSAs for which the type could be determined with at least 95% confidence. Unit bin-width was used and the bins are centered on integer values. The value *k*_1_ = 2 means that if the total GDP increases by e.g. 5% then the share of agriculture decreases by 10%.

In order to illustrate the magnitude of change in share of agriculture, in Fig 2 we show histograms of the parameter *k*_1_. If we rewrite Eq (1) for *g*_0_ = 0 as follows ln*a* = −*k*_1_ *g*, ln*a* = −*k*_1_ln*G*, one can see that *k*_1_ = −1 means that e.g. a 5% increase of total GDP comes along with an increase of agriculture share by the same amount. In case of all considered MSAs, 27 out 126 exhibit *k*_1_ ≤ −0.5. In case of those MSAs where the type was determined with 95% confidence, 10 out of 40 exhibit *k*_1_ ≤ −0.5. Accordingly, the increasing agriculture sector is a non-negligible finding.

**Fig 3:**
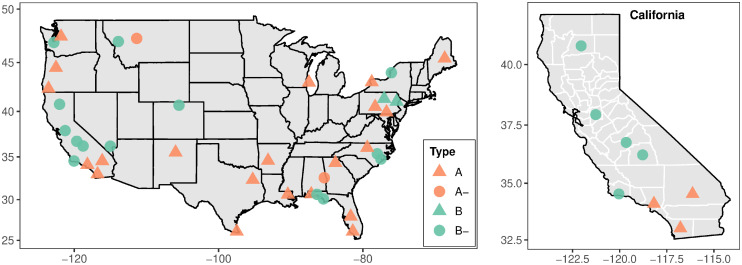
Locations of the MSA and their types at 95% confidence as summarized in Table 2. Top panel: contiguous USA (the black lines delineate states). Bottom panel: the state of California (the white lines delineate counties). The colors distinguish the types A/A^−^ and B/B^−^. The shape of the symbols indicates the direction of development, i.e. triangles for *g* − *g*_0_ > 0 and circles for *g* − *g*_0_ < 0 (“time reversal”).

Fig 3 displays the locations and obtained types of the considered MSAs. Geographically, the result is mixed. Focusing at the 95% confidence level, most MSA are located either at the East or at the West coast. In California, where there are 8 MSAs for which we had suitable data (and the types were determined at high confidence level), 5 of which belong to types with increasing agriculture sectors. While MSAs like Los Angeles and San Diego belong to type A, the conventional model, the three MSAs in the Central Valley are of types B^−^. It is interesting to observe that while some MSAs exhibit a very strong service sector, others engage in a pathway of *agriculture renaissance*—despite reducing groundwater [21]. Many factors in California may contribute to this phenomenon, including ongoing industrialization of the agricultural sector near urban areas [22, 23] and government initiatives promoting organic and other alternative agrifood production [24].

## Discussion

In summary, we have studied the sectoral GDP shares of 126 MSAs in the USA and how those shares change over time. Under relaxed conditions, the employed transfer model allows 6 MSA types, which emerge from the parameter ranges, while being based on the same model and equations. However, the parameters determining the types of the classification are fundamentally different. Consequently, the dynamics are also fundamentally different. From fitting we find that, at the 95% confidence level, 40% of the remaining MSAs display a growing agriculture sector, while more than half of the MSAs follow the consecutive development from agriculture to industry to service.

During the 1980s, job growth in manufacturing, wholesaling, retail, and service industries occurred in the urban peripheries of Los Angeles, suggesting residential movement towards the metropolitan edge and subsequent decentralization of forms [25]. Gordon & Richardson [26] describe it as a dispersed rather than a polycentric metropolis. Between the 1970s and 2010 the household income in Los Angeles developed significantly worse than e.g. in San Francisco [27]. Conventional explanations include the decline of aerospace industry and large population of low-skilled immigrants [27]. A large fraction of employees in the cultural-product industries is found in Los Angeles (together with New York) [28]. Declining (aerospace) industry and strong cultural production, which involves a range of services, fits to the consecutive development of type A.

While Fresno was once a powerhouse of agricultural production, since 2001 its economy has shrunk [29]. High taxes, high business costs, and high levels of crime in combination with a lack of cheap housing and skilled labour have made Fresno less competitive, both within the US and internationally. Cook [29] describes it as “Economic Death Valley” and one of the worst places for businesses. In terms of poverty rates, Fresno had one of the highest among suburban metro areas in 2008 [30]. Michieka & Gearhart [31] find indication for long-term causality running from agricultural abundance to education and long-term causality from oil abundance to education. The declining economy is in agreement with the backward development of type B^−^. Note that this is detected although overall GDP is growing. However, forward relocation from industry to service cannot be easily identified in the literature.

In the traditional development literature, development is associated with a structural transformation in the form of declining agriculture [32]. In contrast, agriculture renaissance is a process often discussed in the context of developing and emerging economies fostering the agriculture sector [33]. On the scale of MSAs and their regions, the implications of re-engagement in agriculture are widely unknown. It is interesting to observe that, on the one hand, the Central Valley exhibits very high agricultural productivity [34] but, on the other hand, includes some of the poorest counties in California.

Agriculture renaissance can be linked to increasing demand for local and regional food [35]. Studies show underlying potential of (peri)urban agriculture to meet such demand [36–38]. Increasingly, (peri)urban agriculture is multifunctional in that it provides multiple services (environmental, social, and economic) to urban inhabitants [39]. Energy use for agriculture and the food system can also be reduced by upscaling (peri)urban agriculture [40]. Share of agriculture GDP may increase in the future with growing role of (peri)urban food production. Another reason for a (seemingly) growing agriculture sector could be the collapse of a local manufacturing sector. A follow-up question could be to which extent the poverty of the central valley is due to agriculture renaissance or if it is rather a process of loss of manufacturing.

Our work can contribute to and inform policy in several ways. First, our model, regression, and classification can help planners and other policy makers understand how the economy of their respective MSA developed in the past. It is likely that in some areas it is not obvious that e.g. the industry sector is over-represented (type C). Second, our work can help identify incentives that direct an MSA’s economy toward a desired future. For instance, development programs could e.g. fund re-education of farmers to find employment in service sectors, and thereby readjust the parameters (1 − *α*)*k*_1_ = *k*_*as*_.

From the methodological point of view, one may criticize that the rather short time series hinders reliable fitting, although by means of bootstrapping we could identify the confident cases. Sources of uncertainty include noise and measurement error in the data, consequent effects on the statistics, and limitations of the model. One could use another parameterization, such as the year as an independent variable or GDP per capita as in [7], instead of the logarithmic total GDP (*g*). Studying the DST model of the shares as a function of the year, we obtain mostly the same types (only 8 out of 126 differ). Regarding the fitting, one could also explore ways of fitting the ternary representation. The DST model has been applied on the country scale [7] and here we transfer it to the city scale. However, going further into the past (e.g. first half of 20th century or the era of industrialization) might require a different model or at least a readjustment of the parameters as profound economic changes took place.

To better understand our results, future research could attempt to establish a connection between the types of development and explanatory properties. With increasing data availability, one could seek to identify changes in development, such as before and after the subprime mortgage crisis of 2007-2010. Another promising direction would be to explore how the model could be used to describe innovation cycles in the context of a hierarchical structure of urban systems [41]. Finally, we note that the land use pendant in the sense of the Hoyt model [42], leads to the question of how—analogously to the economic sectors—land use fractions evolve and if they exhibit similar transition dynamics [43].

## Materials and methods

### Data

We use publicly available data from the US Bureau of Economic Analysis (BEA), an agency of the US Department of Commerce. Using the 2007 North American Industry Classification System (NAICS), this data provides estimated annual GDP for each US MSA disaggregated by industry code. From https://apps.bea.gov/regional/downloadzip.cfm, i.e. “Regional Economic Accounts: Download > Gross Domestic Product (GDP) > CAGDP2: GDP in Current Dollars by County and MSA”, we downloaded the file CAGDP2.zip (access date: 13.01.2021 11:20). The csv-file CAGDP2_MSA_2001_2019.csv includes the MSA data covering the 19 years from 2001-2019.

While the official NAICS hierarchy does not aggregate above the 20 2-digit classification codes, we aggregate to our three economic sectors as follows. For agricultural GDP, *a*, we use the values from “Agriculture, forestry, fishing, and hunting” (NAICS 11). For industrial GDP, *i*, we aggregate the values from “Mining” (NAICS 21), “Utilities” (NAICS 22), “Construction” (NAICS 23), and “Manufacturing” (NAICS 31-33). For service GDP, *s*, we aggregate the remaining NAICS classes 42, 44-45, 48-49, 51, 52, 53, 54, 55, 56, 61, 62, 71, 72, 81. Last we calculate the shares by dividing by the sum over the 3 sectors, e.g. *a* → *a*/(*a* + *i* + *s*). Accordingly, the 3 sectors are normalized to *a* + *i* + *s* = 1. For total urban GDP, *G*, we use the sum of the 3 sectors before normalization. Examples are shown in Fig 1.

Since we study the shares of the total GDP, we had to omit any year for which at least one of the NAICS classes was not available. Moreover, we discarded MSAs with less than 7 years of available data. These constraints restricted our analysis to 126 of the 384 MSAs for which data was sufficient. We apply such strict criteria because we found that ignoring missing values in one or more of the above mentioned NAICS can lead to very different data. The fact that we only consider approximately a third of all MSAs could potentially lead to some sort of bias. However, we find it plausible that the unavailability of data is rather random and independent of any development type. Among those MSAs with adequate data the 3 with the highest GDP are Los Angeles-Long Beach-Anaheim, CA (GeoFIPS 31080), Miami-Fort Lauderdale-Pompano Beach, FL (GeoFIPS 33100), and Seattle-Tacoma-Bellevue, WA (GeoFIPS 42660).

### Dynamical Sector Transfer model (DST model)

We employ a dynamical model that has been applied to characterize the development of sectoral Gross Domestic Product (GDP) composition on the national scale [7]. While originally the considered units were countries, in our application they are cities. The model is inspired by chemical reaction and describes 3 concentrations. In our case the 3 compartments are shares of total urban GDP, namely the aggregated sectors *agriculture* (*a*), *industry* (*i*), and *service* (*s*), which are parameterized against the logarithm of the total urban GDP (*g* = ln*G*) serving as indicator of (economic) development.

**Fig 4:**
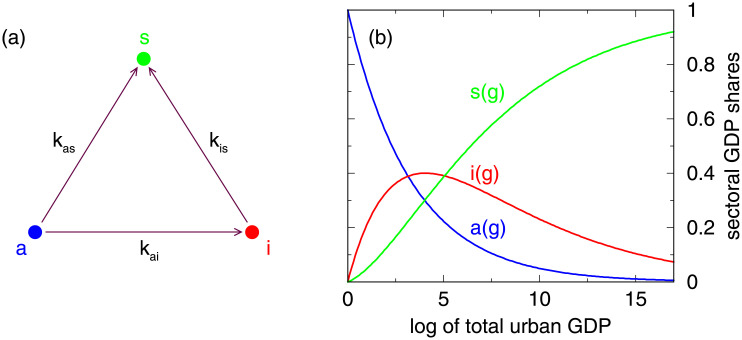
Illustration of the transfer dynamics among urban agriculture, urban industry, and urban service. (a) For illustrative purposes here we use a different set of parameters, namely $\frac{\text{d}a}{\text{d}g}=-k_{ai}a-k_{as}a$, $\frac{\text{d}i}{\text{d}g}=k_{ai}a-k_{is}i$, $\frac{\text{d}s}{\text{d}g}=k_{as}a+k_{is}i$, with *k*_*ai*_ = *αk*_1_, *k*_*is*_ = *k*_2_, and *k*_*as*_ = (1 − *α*)*k*_1_ [7]. (b) Illustrative example of Eqs (1)–(3), i.e. for *k*_1_ = 0.3, *k*_2_ = 0.2, *α* = 0.9, and *g*_0_ = 0.

The sectoral model is given by

$$\begin{array}{ccc} \frac{\text{d}a}{\text{d}g} & = & -k_{1}a \end{array}$$ (4)

$$\begin{array}{ccc} \frac{\text{d}i}{\text{d}g} & = & \alpha k_{1}a-k_{2}i \end{array}$$ (5)

$$\begin{array}{ccc} \frac{\text{d}s}{\text{d}g} & = & \left(1-\alpha \right)k_{1}a+k_{2}i\;, \end{array}$$ (6)

where *k*_1_, *k*_2_, and *α* are parameters. The transfer from agriculture is determined by *k*_1_, which for *α* = 1 goes fully to industry and for *α* = 0 goes fully to service (for 0 < *α* < 1 the transfer is split). The transfer from industry to service is determined by *k*_2_. For *k*_1_ > 0, *k*_2_ > 0, and 0 ≤ *α* ≤ 1 the transfer is directed from agriculture (via industry) to service. Fig 4(a) illustrates the transfer among *a*, *i*, and *s*. It is a multi-compartment model (with 3 compartments) in a closed version since the contents are preserved, i.e. −*k*_1_ + *αk*_1_ + (1 − *α*)*k*_1_ = 0 (factors before *a*) −*k*_2_ + *k*_2_ = 0 (factors before *i*).

The solution to the system of coupled differential equations is given by [7] and leads to the Eqs (1)–(3). An additional parameter *g*_0_ emerges from integration which corresponds to the *g* at which there is solely agriculture. In Eq (2) it becomes clear that *k*_1_ ≠ *k*_2_ must be fulfilled. Moreover, Eq (1) can be rewritten as $a∼G^{-k_{1}}$, i.e. a power-law decay with increasing GDP. Correspondingly, industry in- and decreases approximately following power-laws. For small values of *g* − *g*_0_ we have *i* ≈ *αk*_1_(*g* − *g*_0_) ∼ (*g* − *g*_0_). The maximum of *i* is at $g-g_{0}=\frac{\ln(k_{1}/k_{2})}{k_{1}-k_{2}}$. Fig 4(a) illustrates the transfer between the 3 compartments and Fig 4(b) displays example curves, see also [7, Fig 1].

### Six types

As in [7] we do not constrain the ranges of the 4 parameters (but find *α* > 0 in all cases). Thus, we also allow and can detect dynamics other than the consecutive one (*k*_1_ > 0, *k*_2_ > 0, and 0 ≤ *α* ≤ 1) as illustrated in Fig 4(b). A closer inspection of the system reveals that there are 16 potential cases. However, 10 of them are invalid since at least one of the three quantities is outside of the range [0, 1]. Let us first consider the 8 potential cases for *g* − *g*_0_ > 0. Values *k*_1_ < 0 in Eq (4) lead to a positive derivative $\frac{\text{d}a}{\text{d}g}>0$. Due to the initial condition *a*(*g*_0_) = 1, *a*(*g*) will increase beyond 1 with increasing *g*. This rules out 4 potential cases. Similarly, values (1 − *α*)*k*_1_ < 0 ∧ *k*_2_ < 0 in Eq (6) lead to a negative derivative $\frac{\text{d}s}{\text{d}g}<0$. Due to the initial condition *s*(*g*_0_) = 0, *s*(*g*) will decrease below 0 with increasing *g*. This rules out another potential case and there are 3 remaining cases for *g* − *g*_0_ > 0. Because of symmetry reasons, analogous 3 cases remain for *g* − *g*_0_ < 0, so that in total 6 cases exhibit valid values of *a*, *i*, *s* in at least certain range of *g*. The 6 types are listed in Table 1.

### Non-linear curve fitting

We fit the DST model to the observational data in two steps for each considered MSA. First we note that Eqs (1) and (2) *collapse* if (*g* − *g*_0_) is eliminated, i.e. $\frac{k_{2}-k_{1}}{\alpha k_{1}}i\left(g\right)=(a\left(g\right)-a{\left(g\right)}^{k_{2}/k_{1}})$ [7]. It holds also when both parameters *k*_1_, *k*_2_ are multiplied by the same factor *c*, i.e. $k_{1}^{\prime }=c\;k_{1}$ and $k_{2}^{\prime }=c\;k_{2}$, as it cuts out. This means the relation captures only the fraction of both parameters and we can introduce *β* = *k*_2_/*k*_1_,

$$\begin{array}{c} i\left(a\right)=\frac{\alpha }{\beta -1}(a-a^{\beta })\;, \end{array}$$ (7)

with *β* ≠ 1. It essentially captures which form industry as a function of agriculture follows. For type A and C Eq (7) is a convex function (open to the bottom) and for type B it is a concave one. We use the python scipy.optimize function minimize (0.11.0) with the Limited-Memory Broyden-Fletcher-Goldfarb-Shanno algorithm (L-BFGS-B) to fit Eq (7) to the data. As starting values for *α* and *β* we use random values [0.5, 1.5] and [−1.0, 1.0], respectively. This procedure leads to estimates of *α* and *β*.

In the second step, we use Ordinary Least Squares regression to fit Eq (4) to the agriculture data, i.e. a linear regression to the logarithmic shares. This leads to estimates of *k*_1_ and *g*_0_. The last parameter is obtained by multiplying *k*_1_ with *β*, i.e. *k*_2_ = *k*_1_ *β*.

### Confidence

In order to get an idea about the confidence we employ bootstrapping. We choose bootstrapping because it represents a relatively simply method, given that we are fitting three functions and one of the steps involves non-linear curve fitting.

For a given MSA we construct a new data set with the same sample size by randomly drawing tuples from the original one, whereas repetitions are allowed. Then we apply the regression, obtain parameters, classify the data set, and repeat 100 times.

Detailed results for the examples shown in Fig 1 are listed in Table 3. As confidence we take the highest occurrence, e.g. the development of the Miami MSA (33100) follows with 81% type C. Similar results have been obtained for all 126 considered MSAs and for 40 the confidence level is 95% or higher (80%: 63).

**Table 3:** Bootstrapping results for the example MSAs from Fig 1.

MSA	State	GeoFIPS	Fig 1	Type	A	B	C	C^−^	B^−^	A^−^
Los Angeles-Long Beach-Anaheim	CA	31080	5	A	100	0	0	0	0	0
East Stroudsburg	PA	20700	2	B	0	100	0	0	0	0
Miami-Fort Lauderdale-Pompano Beach	FL	33100	4	C	19	0	81	0	0	0
-	-	-	-	C^−^	0	0	0	0	0	0
Fresno	CA	23420	3	B^−^	0	3	0	0	97	0
Great Falls	MT	24500	1	A^−^	3	0	0	0	1	96

‘Type’ corresponds to the type that has been determined without bootstrapping. The columns ‘A’, ‘B’, …, ‘A^−^’ provide the counts that have been obtained from 100 bootstrapping repetitions. The column ‘Fig 1’ provides the label used therein.
